# Insights into the Endocrine Disrupting Activity of Emerging Non-Phthalate Alternate Plasticizers against Thyroid Hormone Receptor: A Structural Perspective

**DOI:** 10.3390/toxics10050263

**Published:** 2022-05-19

**Authors:** Torki A. Zughaibi, Ishfaq Ahmad Sheikh, Mohd Amin Beg

**Affiliations:** 1 Department of Medical Laboratory Sciences, Faculty of Applied Medical Sciences, King Abdulaziz University, Jeddah 21589, Saudi Arabia; taalzughaibi@kau.edu.sa (T.A.Z.); mbeg@kau.edu.sa (M.A.B.); 2 King Fahd Medical Research Center, King Abdulaziz University, Jeddah 21589, Saudi Arabia

**Keywords:** endocrine disruption, alternate plasticizers, structural binding characterization, thyroid hormone receptor, thyroid dysfunction

## Abstract

Many endocrine-disrupting chemicals (EDCs) have a ubiquitous presence in our environment due to anthropogenic activity. These EDCs can disrupt hormone signaling in the human and animal body systems including the very important hypothalamic-pituitary-thyroid (HPT) axis causing adverse health effects. Thyroxine (T4) and triiodothyronine (T3) are hormones of the HPT axis which are essential for regulation of metabolism, heart rate, body temperature, growth, development, etc. In this study, potential endocrine-disrupting activity of the most common phthalate plasticizer, DEHP, and emerging non-phthalate alternate plasticizers, DINCH, ATBC, and DEHA against thyroid hormone receptor (TRα) were characterized. The structural binding characterization of indicated ligands was performed against the TRα ligand binding site employing Schrodinger’s induced fit docking (IFD) approach. The molecular simulations of interactions of the ligands against the residues lining a TRα binding pocket, including bonding interactions, binding energy, docking score, and IFD score were analyzed. In addition, the structural binding characterization of TRα native ligand, T3, was also done for comparative analysis. The results revealed that all ligands were placed stably in the TRα ligand-binding pocket. The binding energy values were highest for DINCH, followed by ATBC, and were higher than the values estimated for TRα native ligand, T3, whereas the values for DEHA and DEHP were similar and comparable to that of T3. This study suggested that all the indicated plasticizers have the potential for thyroid hormone disruption with two alternate plasticizers, DINCH and ATBC, exhibiting higher potential for thyroid dysfunction compared to DEHA and DEHP.

## 1. Introduction

Phthalate plasticizers are the most common additive compounds used for improving the flexibility and durability of polymer matrices in plastic products, and account for more than 60% of the global plasticizer market [1,2]. The higher molecular weight phthalate plasticizers, such as di(2-ethylhexyl) phthalate (DEHP) are extensively used in polyvinyl chloride (PVC) plastic materials [3,4]. The PVC plastics have vast applications in industrial as well as personal care products, such as automobiles, medical devices and equipment, blood storage bags, surgical gloves, household items, children’s toys, plastic bags, food contact and food packaging materials, cosmetic products, etc., [5,6,7,8]. However, DEHP and other phthalate plasticizers tend to leach out of PVC products over a period of time due to their non-covalent binding with the polymer matrix, leading to their release and ubiquitous presence in the environment [9,10,11,12,13]. As a result, phthalate compounds gain access to human and animal systems and are known to cause hepatotoxic, carcinogenic, reproductive, developmental, neurotoxic, growth-associated, and endocrine system problems, including thyroid problems [2,14,15]. These adverse effects have prompted the European Union and the United States to impose restrictions on the use of several phthalates, including DEHP in many children’s and medical products [16]. In this regard, DEHP has been identified as a “substance of very high concern” under REACH legislation with carcinogenic (group 2B), reproductive toxicity, and endocrine disrupting effects in humans [17]. Similarly, limitations have been put on the percentage of DEHP additive in PVC used in children’s products and medical devices to 0.1% by mass of plasticized material [18]. Due to these stringent regulations on phthalate use and the increased awareness of phthalate toxicity, the demand for a replacement for phthalate plasticizers with non-phthalate alternate compounds, such as diisononyl hexahydrophthalate (DINCH), DEHA acetyl tributyl citrate (ATBC), di-(2-ethylhexyl) adipate (DEHA), etc., has amplified rapidly in the past few years [19,20]. According to a very recent report, the global estimate of the non-phthalate plasticizer market in the year 2021 was USD 3.1 billion and the market size is projected to rise to USD 3.9 billion by the year 2025 [21].

Similar to phthalate compounds, alternate plasticizers also easily leach out from their source material, such as with toys, childcare articles, flooring, furniture, etc., and contaminate the home and office environment, children’s school environment, aquatic environments, sediments, biota, food items, etc., [22,23,24,25,26]. In this regard, alternate plasticizers have been found in school and home dust across the globe, which is attributable to migration from toys, childcare articles, and flooring, furniture, etc., [2]. In addition, the alternate plasticizers DEHA and DINCH were detected in up to 86% of foods (hamburgers, chicken nuggets, fries, chicken burritos, and cheese pizzas) sampled from top-ranked restaurants in Texas, USA [23]. A recent study reported 56% of human serum samples from a US population had detectable levels of DINCH metabolites [27]. In another study, alternate plasticizers have been detected in urinary samples of school kids from many countries, including Saudi Arabia [28].

In view of this, together with the increasing use of non-phthalate plasticizers, con-cerns have emerged regarding the inadequate toxicological data for their safety due to the limited number of studies. Although these were expected to be safe alternatives for phthalate compounds, a few recent studies, including ours, have reported their potential adverse effects on human health [29,30,31]. For example, a recent study showed an association between DINCH exposure and various health problems in pregnant women [32,33]. Similarly, ATBC exposure in mice showed anti-estrogenic as well as anti-androgenic activity. In addition, it also showed adverse effects on ovarian folliculogenesis [34]. Furthermore, metabolites of DEHA were shown to induce cytotoxicity in murine L929 Cell Line [35]. In our recent in silico study, we also reported potential thyroid dysregulation by non-phthalate alternate plasticizers, such as DINCH, DEHA, ATBC, etc., because of interactions with thyroxine-binding globulin [36]. All these above and other limited evidences point to the potential health risks of these emerging environmental contaminants, which is a serious cause of concern.

Thyroid dysfunction constitutes one of the common endocrine diseases in the world [37,38]. The hormones thyroxine (T4) and triiodothyronine (T3) are vital for growth, development, and metabolism. In addition, thyroid hormones are essential components of a cross talk among brain, gonads, sex steroid hormones, and reproductive function—a function which is conserved in almost all vertebrates [39]. Thyroid dysfunction can be due to many factors, such as iodine deficiency and autoimmune diseases, age, disease, etc., [40]. However, environmental factors, including chemical pollutants, are increasingly regarded as contributing factors of thyroid dysfunction because of their effects on the hypothalamic-pituitary-axis and thyroid hormone secretion, and transport and signaling leading to metabolic problems, developmental abnormalities, reproductive outcomes, etc., [40,41]. In this regard, limited reports for alternate plasticizers have also demonstrated their effects on thyroid function. A recent study reported that higher DINCH metabolite (MOiNCH) concentrations in the urine of pregnant women were associated with higher total T3 concentrations, a lower total T4/total T3 ratio, and a lower total T3/free T3 ratio [42]. In other experimental studies, DINCH was reported to cause a significant increase in the weight of the liver and thyroid and hyperplasia/hypertrophy of the thyroid follicles in both male and female rats [43]. The thyroid disrupting potential was also shown by other alternate phthalates, such as DEHA, by inhibiting the proliferation of thyroid-dependent rat pituitary GH3 cells [44]. Taken together, exposure of alternate plasticizers during pregnancy, neonatal life, and adulthood leading to interference of thyroid hormone homeostasis may impair neuro-cognitive development in children and other thyroid hormone-related reproductive and metabolic problems in children and adults.

Very limited toxicity studies are available on alternate non-phthalate plasticizers. The structural binding studies of TRα with DINCH, ATBC, and DEHA are also not available. This is the first study reporting the potential thyroid dysfunction by alternate plasticizers. In view of the potential thyroid related health risks of alternate plasticizers and their largely blank toxicological profiles, it is essential to conduct epidemiological, experimental, and computational studies on these emerging environmental contaminants, especially taking into account human exposure and evidence. The thyroid hormone receptor alpha (TRα) is an essential component of the HPT axis involved in thyroid hormone signaling. Any interference with thyroid hormone signaling is expected to disturb the hemostatic balance of thyroid hormone in the body, with cascading effects on other associated biological pathways.

In the present study, the structural binding characterization of a commonly used phthalate plasticizer, DEHP, and three alternate non-phthalate plasticizers, DINCH, ATBC, and DEHA, was performed against the ligand-binding pocket of TRα using a molecular docking simulation approach. The overall objective of the present investigation was to explore the potential thyroid disrupting activity of the three indicated non-phthalate alternate plasticizers and their comparison with the most commonly used phthalate plasticizer, DEHP.

## 2. Materials and Methods

The molecular structural study on the three emerging alternate non-phthalate origin plasticizers, DINCH, ATBC, DEHA, and the most commonly used phthalate plasticizer, DEHP, against TRα was performed using Schrodinger 2017 suite. The three-dimensional structures of all ligands were retrieved from PubChem compound database (https://pubchem.ncbi.nlm.nih.gov/) (accessed on 15 November 2021). All these plasticizer ligands were subjected to structural binding characterization using Schrodinger 2017 suite with Maestro 11.4 as the graphical user interface (Schrodinger, LLC, New York, NY, USA, 2017). The methodology is described below.

### 2.1. Protein Preparation

The crystal structure of human TRα (PDB code: 2H79) in complex with the native ligand, T3, solved at a very high resolution of 1.87 Å, was chosen for downstream com-putational study. The protein preparation wizard workflow of Schrodinger Glide for molecular simulation studies (Schrodinger suite 2017-4; Schrodinger, LLC) automatically imports structure coordinates from Protein Data Bank (PDB; http://www.rcsb.org/) (accessed on 10 November 2021). The retrieved crystal complex was then further processed and prepared using a protein preparation wizard. It further added missing hydrogen atoms and corrected the metal ionization states. It also enumerated the bond order to HET groups and removed co-crystallized water molecules. It further capped the N-terminal of protein with ACE (N-acetyl) and C-terminal with NMA (N-methyl amide). In addition, it also highlighted amino acid residues which had multiple occupancies or missing atoms. Moreover, the protein preparation wizard also determined the most likely ligand protonation state. Furthermore, for the Histidine residue, the optimal protonation state was determined as well. Finally, the hydrogen bond network optimization by means of a systematic, cluster-based approach, followed by energy minimization was performed. The bound native ligand, T3, in the imported PDB was automatically prepared during the protein preparation wizard step by fixing its formal charges and bond order. Further ionization and tautomeric states were also prepared during this step by running Epik. 

### 2.2. Ligand Preparation

**Figure 1:**
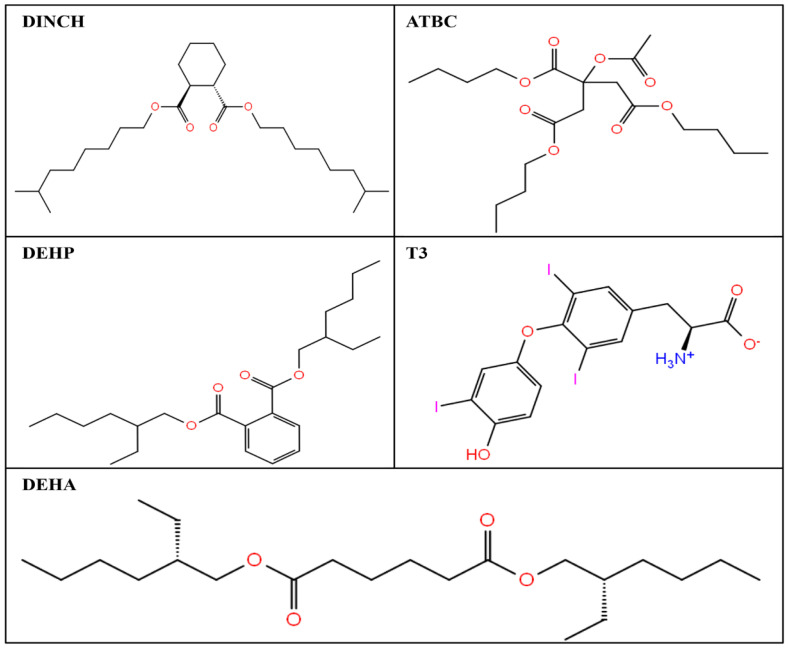
Two-dimensional structures of diisononyl hexahydrophthalate (DINCH), acetyl tributyl citrate (ATBC), di(2-ethylhexyl) phthalate (DEHP), thyroid receptor (TRα) native ligand, triiodothyronine (T3), and di-(2-ethylhexyl) adipate (DEHA).

The LigPrep module from Schrodinger suite was employed to prepare all the downloaded ligands for simulation studies (Schrodinger 2017: LigPrep, Schrodinger, LLC). This is an efficient module and prepares one ligand in approximately one second for downstream computational processes. The PubChem compound identity of all the downloaded ligands (DEHP, DINCH, ATBC, and DEHA) are mentioned in Table 1. This module produces energy-minimized, accurate three-dimensional structures for the ligands and also applies filters to remove those compounds which fail to meet the user-specified criteria. The LigPrep module eliminate mistakes in ligands and corrects Lewis structures. Furthermore, it also produces structural and chemical diversity, such as various stereoisomers, ring conformations, tautomeric and ionization states, etc. from a given input ligand structure. The two-dimensional structures of all the indicated ligands are presented in Figure 1.

### 2.3. Induced Fit Docking

The Schrodinger’s Induced Fit Docking (IFD) module was used to perform the docking of all the indicated plasticizers and T3 in the TRα ligand-binding pocket as described in our previous study [22]. The IFD docking is not rigid as it induces flexibility in the protein receptor ligand-binding site as well as the ligand. The Schrodinger’s Glide and Refinement module in Prime were employed to develop and validate IFD protocols for accurate prediction of receptor ligand-binding poses and the associated changes in the ligand-binding pocket of the protein receptor. Briefly, the first step in IFD execution was the grid generation at the TRα native ligand, T3 binding site. Then the constrained minimization of the protein receptor was performed using the Protein preparation step with RMSD cutoff of 0.18 Å. This was followed by initial Glide docking using a softened potential and optional side chain removal for all the ligands. The total number of maximum docking poses which were retained by default were twenty. Then, side chains were predicted in amino acids within 5 Å distance in each receptor-ligand complex for any pose followed by minimization. The ligand was also minimized for each complex (receptor-ligand) pose. It was further followed by Glide re-docking and IFD score estimation. Likewise, extended sampling protocol was also performed. In addition, the TRα native ligand, T3, was also subjected to IFD.

### 2.4. Binding Affinity Calculations

The Prime module of Schrodinger 2017 with MMGB-SA function was used to estimate the binding affinity of all the plasticizer ligands against the TRα binding pocket. The binding energy (ΔG_Bind_) values suggest how stably the ligand is bound to protein. The Prime MMGB-SA produces a lot of energy properties, reporting the energies for receptor, ligand, complex structures, and energy differences relating to strain and binding. The main five energy calculations done in MM-GBSA are: Optimized free ligand (=“Ligand”), Optimized free receptor (=“Receptor”), Optimized complex (=“Complex”), Ligand from minimized/optimized complex, and Receptor from minimized/optimized complex. These energy values are used to calculate receptor strain, Ligand strain, and MMGBSA dG Bind energies. The binding free energy (Prime MMGBSA DG bind) is estimated using the following equation:

ΔG_bind_ = E_complex_ (minimized) − [E_ligand_ (minimized) + E_receptor_ (minimized)]

where ΔG_bind_ is binding free energy and E_complex_ (minimized, E_ligand_ (minimized), and E_receptor_ (minimized) are minimized energies of the receptor-ligand complex, ligand, and receptor, respectively.

## 3. Results

**Figure 2:**
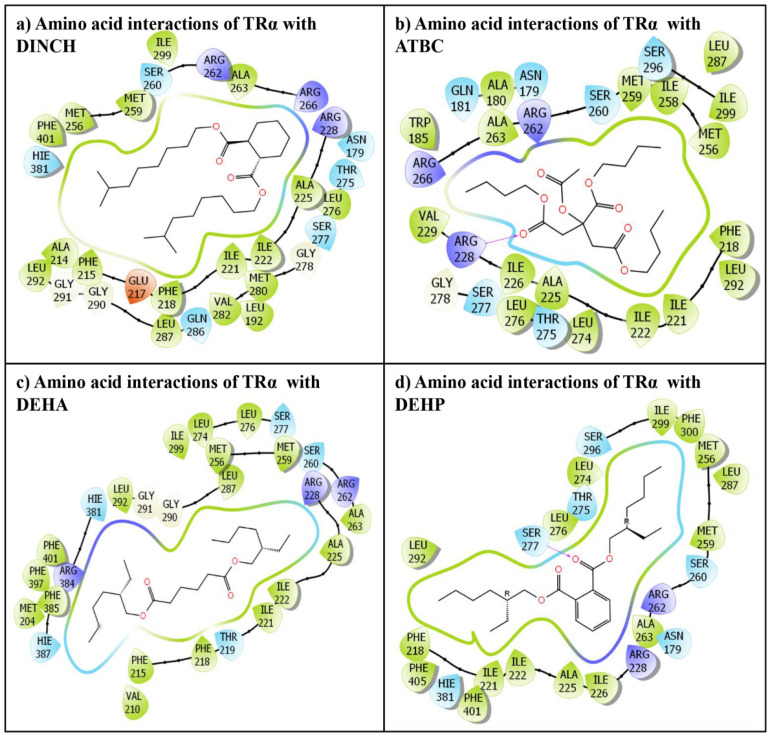
The molecular interactions of non-phthalate plasticizers (**a**) diisononyl hexahydrophthalate (DINCH), (**b**) acetyl tributyl citrate (ATBC), (**c**) di-(2-ethylhexyl) adipate (DEHA), and (**d**) di (2-ethylhexyl) phthalate (DEHP) with residues lining the thyroid receptor (TRα) ligand-binding pocket.

**Figure 3:**
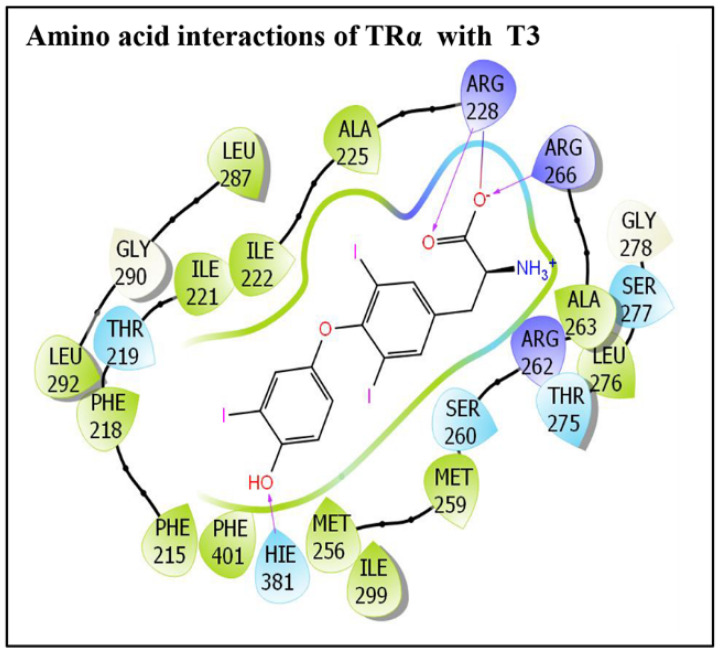
The molecular interactions of a triiodothyronine (TRα) native ligand and triiodothyronine (T3) with residues lining TRα ligand-binding pocket.

The commonly used the phthalate plasticizer, DEHP, and the three emerging non-phthalate alternate plasticizers, DINCH, ATBC, and DEHA, docked successfully in the TRα ligand-binding pocket. The IFD approach placed all the ligands tightly, suggesting their stable binding in the TRα binding pocket. Several docking display poses were generated; however, only the best ranking poses were selected and taken forward for further structural characterization analysis. Similarly, the native ligand, T3, was also placed successfully in TRα ligand-binding following IFD, and again the best ranking pose was selected for the native ligand too. The selected poses displaying several amino acid residue interactions with all the indicated ligands are presented (Figure 2). Likewise, the selected docking display of TRα native ligand, T3, is also presented (Figure 3). The alternate plasticizers displayed interactions with 27–30 amino acid residue lining the TRα ligand binding pocket (Figure 2a–c); however, the phthalate plasticizer, DEHP, exhibited interactions with 24 amino acid residue interactions (Figure 2d).

### 3.1. IFD of Phthalate Plasticizer Ligand, DEHP, with TRα

The docking complex of DEHP-TRα exhibited numerous interactions with several TRα amino acid residues. In total, 24 TRα amino acid residues were engaged in molecular interactions, such as hydrogen bonding, van der Waals, hydrophobic interactions, etc. with DEHP. The amino acid residues engaged in interactions were: Asn-179, Phe-218, Ile-221, IIe-222, Ala-225, Ile-226, Arg-228, Met-256, Met-259, Ser-260, Arg-262, Ala-263, Leu-274, Thr-275, Leu-276, Ser-277, Leu-287, Leu-292, Ser-296, Ile-299, Phe-300, His-381, Phe-401, and Phe-405. In addition, one hydrogen bonding interaction was also displayed by Ser-277 (Figure 2d). Furthermore, one hydrogen bond interaction was also displayed by Ser-277.

Likewise, the native ligand, T3’s, molecular interactions with TRα are shown (Figure 3). In total, 23 TRα amino acid residues were engaged in molecular interactions with T3, i.e., Phe-215, Phe-218, Phe-219, Ile-221, IIe-222, Ala-225, Arg-228, Met-256, Met-259, Ser-260, Arg-262, Ala-263, Arg-266 Thr-275, Leu-276, Ser-277, Gly-278, Leu-287, Gly-290, Leu-292, Ile-299, His-381, and Phe-401. Furthermore, T3 also exhibited three hydrogen bond interactions, each with Arg-228, Arg-266, and His-381. In addition, one salt bridge interaction is also exhibited by Arg-228 (Figure 3).

Additionally, other estimated parameters such as IFD, Dock score, and Glide score essential for structural binding analysis of the phthalate plasticizer, DEHP, and the TRα native ligand, T3 are also presented (Table 1). Furthermore, binding energy which is another crucial parameter for structural binding characterization was also estimated and the values are presented (Table 1). The estimated binding energy values for the native ligand, T3, and DEHP were very close. Additionally, the overlap in TRα interacting amino acid residues between DEHP and native ligand was about 78%.

### 3.2. IFD of Non-Phthalate Alternate Plasticizers Plasticizers, DINCH, ATBC, and DEHA, with TRα

The new emerging non-phthalate plasticizers displayed several molecular interactions with 27–30 amino acid residues. The docking display pose of DINCH exhibited 30 amino acid residues engaged in various molecular interactions with TRα (Figure 2a). Furthermore, the comparison between the docking poses of the native ligand, T3, and DINCH revealed approximately 96% overlap in amino acid interactions. Moreover, several other molecular interactions were also observed in the DINCH-TRα complex due to additional amino acid residues, i.e., Ala-179, Leu-192, Ala-214, Glu-217, Met-280, Val-282, Gln-286, and Gly-291 (Figure 2a). 

Likewise, the ATBC-TRα docking display pose exhibited 27 amino acid residues of TRα engaged in various molecular interactions (Figure 2b). The comparison between the docking poses of the native ligand, T3, and ATBC-TRα revealed about 78% overlap in amino acid interactions. However, several other molecular interactions were also observed in ATBC-TRα complex due to additional amino acid residues, i.e., Asn-179, Ala-180, Gln-181, Trp-185, Ile-226, Val-229, Ile-258, Leu-274, and Ser-296. Furthermore, one hydrogen bonding interaction was also displayed by Arg-228 (Figure 2b). 

Similarly, DEHA-TRα docking display pose exhibited 28 amino acid residues of TRα engaged in various molecular interactions (Figure 2c). The comparison between the docking poses of the native ligand, T3, and DEHA-TRα revealed approximately 87% overlap in amino acid interactions. However, several other molecular interactions were also observed in DEHA-TRα complex due to additional amino acid residues, i.e., Met-204, Val-210, Leu-274, Gly-291, Arg-384, Phe-385, His-387, and Phe-397 (Figure 2c). 

## 4. Discussion

Phthalate plasticizers, especially DEHP, have been shown to affect the hypothalamic-pituitary-thyroid axis and perturb the thyroid hormone homeostasis in epidemiological studies in humans [42,45] and in experimental laboratory animal and in vitro studies [40,46,47]. Alternative plasticizers are increasingly replacing phthalates in commercial and household products, such as food packaging, medical devices, cosmetic products, sealants, carpet, plastic tubing, fabrics, children’s toys, vinyl flooring, and several other applications [19]. However, concerns regarding these replacement plasticizers have been raised as sufficient evaluation of their toxicity and health risks is not available and it is a challenge to consider them as safe for human-related household and commercial use [2].

The main objective of this study was to investigate, characterize, and compare the structural binding interactions of a very commonly used phthalate plasticizer, DEHP, and three emerging alternate non-phthalate origin plasticizers, DINCH, ATBC, and DEHA in the TRα ligand-binding pocket. The structural binding characterization was aimed to gain molecular insights into the potential endocrine disrupting activity of the indicated com-pounds on TRα signaling. This study used the IFD approach to execute docking experiments for the indicated plasticizers in the TRα ligand-binding pocket. The structural binding analysis results revealed that the DEHP and the alternate non-phthalate plasticizers (DINCH, ATBC, and DEHA) formed stable and successful binding in the TRα ligand-binding site. The binding parameters, such as Dock score, glide score, IFD score, and the binding energy values, also indicated stable and good quality docking complexes for all the plasticizer ligands considered for this study. Various molecular interactions, such as pi-pi interactions, hydrogen bonds, and salt bridge interactions displayed by these ligands contributed to the stability of these complexes. The comparative analysis of the selected docking poses of all the individual four plasticizer complexes with that of TRα native ligand, T3, indicated a 90–100% overlap with the TRα ligand-binding pocket interacting with amino acid residues. In addition, the estimated binding energy values for all the indicated plasticizers were either similar to or higher than the values estimated for the TRα native ligand, T3. The binding energy values of phthalate plasticizer DEHP were similar to the TRα native ligand, T3. However, the binding energy values for DINCH and ATBC were higher than the TRα native ligand, T3. When compared to DEHP, the binding energy values for DINCH and ATBC were higher, 19% and 7%, respectively. In addition, the percentage overlap of amino acid residues interacting in the ligand-binding pocket of TRα was higher for DINCH (96%) compared to that of DEHP (78%); the percentage for ATBC (78%) was similar to that of DEHP. As mentioned above, DEHP is a known disruptor of thyroid function [40,42,45,47]. Therefore, on a preliminary basis, based on the comparative binding energy values and the number of amino acid interactions with TRα, DINCH and ATBC may be considered as potential thyroid disruptors. The binding energy value for DEHA was lower than DINCH and ATBC but was similar to that of DEHP and the TRα native ligand, T3. In addition, the percentage overlap of amino acid residues interacting in the ligand-binding pocket of TRα was higher for DEHA (87%) compared to that of DEHP. Hence, based on similarities in the structural binding of DEHA and DEHP, DEHA may also be considered as a potential thyroid disruptor. Taken together, on a preliminary basis, the three indicated plasticizers and commonly used phthalate compound, DEHP, may interfere with thyroid hormone signaling and thus lead to thyroid gland dysfunction. The absolute estimated binding affinity values may not necessarily match with actual experimental binding affinities values. Nevertheless, the ranking of the ligands based on our calculations (MMGBSA DG Bind) is expected to match reasonably well to the ranking trend based on experimental binding affinity.

Very limited studies are available on the structural binding aspects of the phthalate plasticizer, DEHP, against the TRα ligand-binding pocket. A recent study [46] reported in silico characterization of DEHP and its metabolites with TRα and DEHP was found to not bind to TRα but to its monoester, MEHP, and hydroxylated and oxidative metabolites, 5-OH-MEHP and 5-oxo-MEHP, closely imitated the binding mode in the TRα binding pocket. In the present study, we found that DEHP binds TRα with binding energy that was similar to that of native ligand, T3. The differences in the two studies may be due to the two different molecular docking platforms used. However, our results on molecular docking of DEHP are supported by the results from this former study for DEHP metabolites [46]. Further, using a T-screen assay—a thyroid hormone-dependent rat pituitary tumor cell growth assay—Kambia et al. reported that MEHP and 5-OH-MEHP induced concentration dependent agonist activity in the cells stimulating cell growth, which was synergically enhanced by the addition of T3 [46]; however, 5-oxo-MEHP showed antagonistic and cytotoxic activity. In another study involving TR reporter gene assays using a recombinant Xenopus laevis cell line, DEHP exhibited T3-antagonistic activity [48].

Available epidemiological and laboratory animal studies have reported the association of DEHP exposure with thyroid dysfunction. In this regard, urinary MEHP concentrations had a negative relationship with total T4 in pregnant women, even during early pregnancy when a fetus is more vulnerable to developmental problems [49]. Meta-analysis of epidemiological data showed that early life exposure of DEHP and its metabolites, MEHP and 5-OH-MEHP, was associated with a decrease in T4 and total T4, and an increase in thyroid-stimulating hormone, suggesting effects on thyroid function in children, adults, and pregnant women [45,50]. A recent study reported that higher DEHP metabolite concentrations in the urine of pregnant women were associated with lower free T4 concentrations and higher thyroid stimulating hormone/free T4 ratio [42]. Conversely, urinary DEHP metabolites were positively associated with maternal plasma total T4 and decreased maternal free T4, and thyrotropin during pregnancy [51]. Nevertheless, the early exposure of phthalates may have a potential impact on organogenesis, including neurodevelopment in children, as the thyroid hormones are critically essential for the nervous system during the fetal and early neonatal life [52]. DEHP has also been shown to induce thyroid toxicity and perturb the homeostasis of thyroid hormones in laboratory animals [47,53]. In rats, DEHP caused interference in the hypothalamic-pituitary-axis and lowered the concentrations of free T3, total T3, free T4, and total T4 along with protein and mRNA levels of thyroid stimulating hormone [47]. In another study in rats [53], along with lowering of T4 and increasing of T3, disruption of the redox status, accumulation of malondialdehyde, and depletion of reduced glutathione was found together with histological changes in thyroid follicles [53]. Similar effects have been reported in several other studies [40,54]. In addition, the DEHP interactions with other receptors such as ERα, ERβ, and PPARγ are reported in detail [55,56,57].

The structural binding studies of TRα with alternate non-phthalate plasticizers involving DINCH, ATBC, and DEHA have not been reported to the best of our knowledge. However, a quantitative, high-throughput screening (Tox21) study of 3000 environmentally relevant chemicals against a panel of 10 human nuclear receptors, including TRβ (TRα not tested) for identifying potential agonists and antagonists, resulted in inconclusive results for ATBC against TRβ [58]. Similarly, ATBC was not found to stimulate the transcription of a number of nuclear receptors including TRβ, and very limited information is available for its thyroid disruption effects [59]. Recently, in a reporter assay, DINCH did not show any effect on the activity of human nuclear receptors ERα, ERβ, AR, PPARα, and PPARγ (TRα not tested), but endogenous primary and secondary metabolites of DINCH resulted in activation of all these receptors [60]. Epidemiological studies have shown that DINCH metabolites were detected alongside phthalate metabolites in human urine samples worldwide [14]. Relating to thyroid function, urinary levels of a DINCH metabolite (MOiNCH) were positively associated with total T3 concentrations and a lower total T4/total T3 ratio in women [42]. Relating to the effects on other body systems, urinary metabolites of DINCH were associated with inflammation-derived 8-iso-PGF2α, an oxidative stress and inflammation-related molecule in pregnant women [33]. DINCH may also adversely affect gestational hormones, with potential for gestational age and fetal sex-specific associations such as urinary DINCH metabolites found positively associated with total serum estrogens—2.4% higher with every two-fold increase in metabolites [32]. In another recent study [27], the serum testosterone concentrations were negatively associated with urinary DINCH metabolites.

Some experimental laboratory animal studies provide support indirectly to our results of the potential effects of DINCH on thyroid function. DINCH was considered as a hazard for endocrine activity, based on the effects on the thyroid glands in rats in several studies together with decreased anogenital distance in male offspring and a decreased anogenital index observed in male and female offspring [61]. DINCH exposure resulted in increased weight of the liver and thyroid, in addition to hyperplasia/hypertrophy of the thyroid follicles in male and female rats, and increased testes weight in male rats [43]. Relating the importance of these studies to human thyroid function, the USEPA risk assessment forum considers non-cancer and cancer thyroid effects as a result of disruption of the hypothalamic-pituitary-thyroid axis as a relevant non-cancer and cancer health hazard to humans [43]. In other studies, prenatal exposure of DINCH was associated with premature aging of testes, impaired liver metabolic capacity, and exhibited anti-androgenic effects (decrease of anogenital distance in males) in rats [62,63]. In addition, prenatal and lactational exposures of DINCH were associated with dysregulation of estrogen signaling gene expression in rat testes [64]. Exposure of DINCH in zebra fish caused hatching delays and disturbed the gene expression for stress response [65]. In addition, the exposure caused lipid accumulation in various parts of the body, affected the expression of genes for lipid transport and cholesterol biosynthesis, and impacted the locomotor activity of larvae. In vitro cell culture studies using the male germ spermatogonial cell line (C18–4), a Sertoli cell line (TM4), and two steroidogenic cell lines (MA-10 Leydig and KGN granulosa) have also shown that exposure with MEHP, DINCH, and DEHA resulted in distinct phenotypic effects in all cell lines, in addition to cytotoxicity, oxidative stress effects, mitochondrial activity, and lipid droplet effects [4]. DINCH treatment was also associated with cytotoxicity in kidney cells and oxidative DNA damage in liver cells [66]. DINCH also induced cytotoxic effects in L929 murine cells at biologically relevant concentrations [35]. The metabolites for DINCH were found to be more cytotoxic. Although DEHA did not show cytotoxicity, its metabolites induced a cytotoxic effect [35]. In another recent study, DINCH disrupted steroidogenesis in the H295R assay by inducing an increase in estradiol synthesis [31].

Similar to DINCH, docking and experimental studies on the toxicity profile of ATBC are limited, especially with regard to thyroid function. In one study, ATBC did not show any stimulatory effect on human TR in a luciferase reporter study [67]. However, it showed antiestrogenic and antiandrogenic activity using luciferase reporter assays, HeLa9903 and VM7-Luc cell cultures, induced developmental problems in frog embryo teratogenicity assays, and increased mineralocorticoids in human adrenal H295R cells [30,59,67,68]. Even at a lower dose, ATBC was shown to have detrimental effects on ovarian folliculogenesis and increased apoptotic changes in isolated mouse follicles and the count of static follicles [34]. ATBC has been shown to be a potent inducer of SXR transcription in rabbits, rats, mice, dogs, and humans [59]. ATBC also exerted estrogenic effects, androgenic effects, higher CYP3A4 gene expression, and acted as an SXR agonist; thus, it may alter the metabolism of endogenous steroid hormones [62]. Previously, ATBC has been shown to increase liver weight, bioaccumulation, and abnormal central nervous system function in rats [69]. ATBC caused reproductive/developmental effects at relatively high doses in rats [70].

The thyroid disrupting potential of DEHA was shown in vitro by inhibition of the proliferation of thyroid-dependent rat pituitary GH3 cells [44]. In an earlier study, DEHA exposure prolonged the gestation period and increased postnatal death in rats. In addition, the study revealed a nonrecoverable decrease in offspring body weight causing developmental toxicity [71]. DEHA also reduced body weight gain in rats, reduced mean pup weight in new born rats, induced estrogenic and anti-androgenic effect in male rats, increased follicle atresia in rat ovaries, and decreased sex steroid-regulated gene expression and sexual behavior in male rats [62]. DEHA also caused liver tumors in mice, fetal toxicity in rats, and suppressed the neonatal brain expression of grn in males and p130 in females. In addition to the effects on the hypothalamus, DEHA also caused ovarian follicle atresia and prolonged estrus cycle in females, thus affecting reproductive functions [69,72,73]. 

In summary, our study shows that limited reports are available regarding the toxi-cological profile of alternate plasticizers, especially in relation to thyroid dysfunction. The global use of alternate plasticizers is increasing at a remarkable speed, and that is an indication of the potential magnitude of this emerging environmental problem in relation to human health. Therefore, based on our preliminary molecular docking results of DINCH, ATBC, and DEHA against TRα, the notion of these plasticizers being safe alternatives for DEHP may be taken very cautiously. We suggest further epidemiological, in vivo animal, and in vitro studies for a deeper understanding of the effect of alternate plasticizers against thyroid function and other systems. 

## 5. Conclusions

Increasing global use of alternate phthalate plasticizers and limited studies about their toxicity profile and safety assessment raises serious concerns regarding their impact on human and animal health. The results of our study revealed that the commonly used emerging non-phthalate plasticizers, DINCH, ATBC, and DEHA, and the widely used phthalate plasticizer DEHP, show commonality in interacting amino acid residues in the TRα ligand-binding pocket with that of the TRα native ligand, T3, indicating successful and stable ligand-complex formation. Furthermore, the binding energy values were highest for DINCH followed by ATBC, and the values were higher than those for the TRα native ligand, T3, and DEHP, suggesting their thyroid dysfunction potential. The remaining non-phthalate plasticizer showed binding energy values similar to the TRα native ligand, T3, and DEHP. Hence, the DEHA could also potentially act as a thyroid disruptor. Thus, all the three alternate plasticizers i.e., DINCH, ATBC, and DEHA have the potential to disrupt thyroid signaling, which may result in alteration of thyroid hormone homeostasis, leading to thyroid-related adverse effects on health. In view of our results, more studies are warranted to obtain insights into the thyroid dysfunction potential of this emerging group of plasticizers, which are considered safe replacements for phthalate plasticizers.

## Figures and Tables

**Table 1:** Structural binding indices of plasticizers diisononyl hexahydrophthalate (DINCH), acetyl tributyl citrate (ATBC), di-(2-ethylhexyl) adipate (DEHA), di (2-ethylhexyl) phthalate (DEHP), a thyroid receptor (TRα) native ligand, and triiodothyronine (T3).

Ligand	Number of Interacting TRα Residues	Percentage of Interacting Residues Common with Native Ligand (%)	IFD Score	Docking Score (Kcal/mol)	Glide Score (Kcal/mol)	MMGB-SA (Kcal/mol)
DINCH	30	95.45	−563.87	−9.53	−9.53	−156.49
ATBC	27	78.26	−564.56	−8.89	−8.89	−140.29
DEHA	28	86.95	−564.36	−7.96	−7.96	−130.04
DEHP	24	78.26	−562.08	−8.64	−8.64	−131.67
T3	23	100	−564.42	−9.44	−9.44	−133.53
